# A Molecule of the Viridomycin Family Originating from a *Streptomyces griseus*-Related Strain Has the Ability to Solubilize Rock Phosphate and to Inhibit Microbial Growth

**DOI:** 10.3390/antibiotics10010072

**Published:** 2021-01-14

**Authors:** Hanane Hamdali, Ahmed Lebrihi, Marie Carmen Monje, Ahmed Benharref, Mohamed Hafidi, Yedir Ouhdouch, Marie Joëlle Virolle

**Affiliations:** 1 Laboratory of Biotechnology and Valorization of Plant Genetic Resources, Faculty of Sciences and Techniques, University of Sultan Moulay Slimane, P.O. Box 523, Beni-Mellal 23000, Morocco; hamdali_hanane@yahoo.fr; 2 INPT, Laboratoire de Génie Chimique, UMR 5503 (CNRS/INPT/UPS), École Nationale Supérieure d’Agronomie de Toulouse, 1, Avenue de l’Agrobiopôle, B.P. 107, F-31 326 Castanet-Tolosan CEDEX, France; ahmed.lebrihi@ensat.fr (A.L.); monje@pop.ensat.fr (M.C.M.); 3 Laboratoire of Microbial Biotechnologies, Agrosciences and Environment, Université Cadi Ayyad (UCA), Boulevard Prince My Abdellah B.P. 2390, Marrakech 40000, Morocco; benharref@uca.ac.ma (A.B.); hafidi.ucam@gmail.com (M.H.); youhdouch@gmail.com (Y.O.); 4 AgroBioSciences Program, Mohammed VI Polytechnic University (UM6P), Benguerir 43150, Morocco; 5 CEA, CNRS, Institute for Integrative Biology of the Cell (I2BC), Université Paris-Saclay, 91198 Gif-sur-Yvette, France

**Keywords:** *Streptomyces griseus*, viridomycin, antimicrobial activity, rock phosphate solubilization

## Abstract

Some soil-borne microorganisms are known to have the ability to solubilize insoluble rock phosphate and this process often involves the excretion of organic acids. In this issue, we describe the characterization of a novel solubilizing mechanism used by a *Streptomyces* strain related to *Streptomyces griseus* isolated from Moroccan phosphate mines. This process involves the excretion of a compound belonging to the viridomycin family that was shown to play a major role in the rock phosphate bio weathering process. We propose that the chelation of the positively charged counter ions of phosphate constitutive of rock phosphate by this molecule leads to the destabilization of the structure of rock phosphate. This would result in the solubilization of the negatively charged phosphates, making them available for plant nutrition. Furthermore, this compound was shown to inhibit growth of fungi and Gram positive bacteria, and this antibiotic activity might be due to its strong ability to chelate iron, a metallic ion indispensable for microbial growth. Considering its interesting properties, this metabolite or strains producing it could contribute to the development of sustainable agriculture acting as a novel type of slow release bio-phosphate fertilizer that has also the interesting ability to limit the growth of some common plant pathogens.

## 1. Introduction

Phosphorus (P) is often scarce in natural soils and its low availability limits plant growth and thus agricultural yields [1,2]. To solve this problem, agricultural soils are amended with soluble phosphate. However, the generation of soluble phosphate from natural rock phosphate (RP) is expensive and highly polluting and alternatives should be found. Natural ground RP was used in traditional agriculture with limited success because of its poor solubility. Interestingly several reports in the literature mentioned that some soil-borne microorganisms are able to solubilize mineral phosphates and are thus of great interest to enhance P availability for plant nutrition [2,3]. The introduction of such microorganisms in fields might constitute a way to reduce soluble phosphate fertilizer input [1,2,4]. The commercially available poorly soluble RP is mainly a calcium hydroxyapatite [Ca_10_ (PO_4_)_6_ OH] but calcium could be substituted by other cations such as Fe^3+^, Al^3+^, Na^+^, and Mg^2+^ ions [5]. Numerous reports in the literature mention that most Phosphate Solubilizing Microorganisms (PSM) dissolve insoluble mineral phosphates via secretion of organic acids [6]. In addition, some PSM can contribute to plant health via the production of bioactive substances, limiting the growth of some specific plants pathogens [7,8]. Their use could thus reduce the excessive input of chemical pesticides that are known to be toxic to human health [9]. Among these PSM, *Actinobacteria* [10,11,12,13,14] are of special interest. These filamentous and spore-forming bacteria contribute to soil fertility through their valuable ability to decompose soil organic matter. They also contribute to plant health and fitness thanks to their ability to produce molecules able to limit the growth of devastating bacterial or fungal phytopathogenic agents [15,16]. We previously isolated from Moroccan phosphate mine and characterized a *Streptomyces griseus*-related strain able to efficiently solubilize RP [10] as well as to produce antifungal and anti-bacterial metabolites [11,12]. In this issue we demonstrated that the RP solubilization ability of this strain was due to excretion of a molecule of the viridomycin family. This molecule can allow the slow release of the phosphate of RP via its ability to chelate the positively charged counter ions of the phosphate constitutive of RP.

## 2. Results

### 2.1. Detection and Purification of Bio-Active Fractions

**Figure 1:**
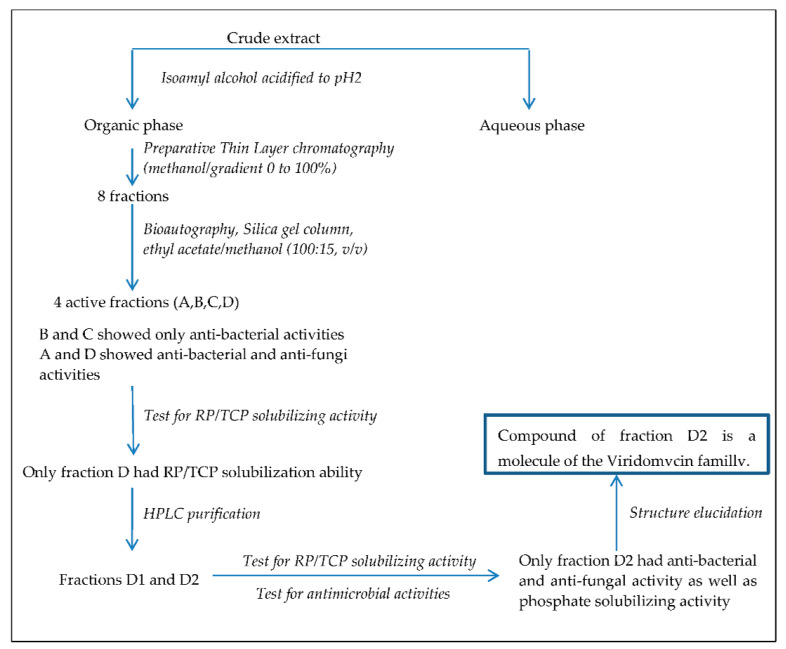
Schematic representation of the workflow used to purify the molecule of the viridomycin family possessing both phosphate-solubilizing and antimicrobial activity. RP and TCP stand for Rock Phosphate and Tri Calcium Phosphate, respectively. HPLC stands for High Performance Liquid Chromatography.

The cell-free supernatant of 3 L cultures of the *Streptomyces griseus*-related strain grown as described in Materials and Methods was extracted with isoamyl alcohol. The resulting greenish organic phase was concentrated to dryness (1.65 g), dissolved in 2 mL of methanol as a crude extract, and analyzed by Thin Layer Chromatography (TLC). After migration and revelation, four active spots were detected by bio autography on the TLC plates. The four fractions designated as A, B, C, and D showed Rf values of 0.9, 0.45, 0.37, and 0.3, respectively. A and D spots showed both antibacterial and antifungal activities against the tested micro-organisms, while B and C showed only antibacterial activity (see material and methods; (Figure 1). Subsequently, the crude extract (275 mg/mL) was deposited on preparative silica gel plates in order to collect the A and D fractions in sufficient quantity. We obtained 115 mg/mL of fraction A (41.8% of crude extract) and 98.5 mg/mL of fraction D (35.8% of crude extract) (Figure 1).

### 2.2. Determination of the Mineral Phosphate-Solubilizing Abilities of the Bio-Active Fractions

In order to determine the mineral phosphate-solubilizing abilities of the bio-active fractions, 500 µL of active fractions A and D at a final concentration 98.5 mg/mL were checked for their ability to solubilize RP and TCP (Tri-calcium phosphate, Ca_3_ (PO_4_)_2_) as described in material and methods (Figure 1). The results demonstrated that only fraction D (greenish color) showed an ability to solubilize RP as well as TCP. The concentrations of solubilized phosphate from RP and TCP were 63.7 µg/mL and 135.2 µg/mL, respectively.

### 2.3. HPLC Analysis of the Bio-Active Fraction D that Also Bears Mineral Phosphate-Solubilizing Ability

**Figure 2:**
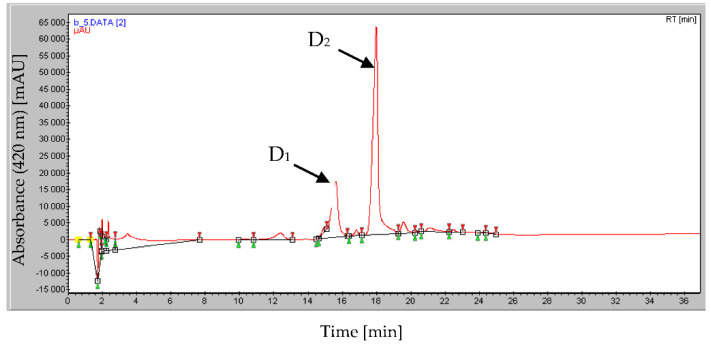
HPLC analysis of the fraction D bearing both antibiotic and rock phosphate-solubilizing activity. This fraction contains two main peaks, D_1_ (Rf = 15.62) and D_2_ (Rf = 17.99 min).

The HPLC chromatogram of the active greenish fraction (D) revealed several peaks. The retention time of the main peaks D_1_ and D_2_ was 15.62 and 17.99 min, respectively (Figure 2).

These sub-fractions were checked again for their ability to solubilize mineral phosphate and for their antibiotic activity against *Micrococcus luteus* ATCC 381 (ML), *Bacillus subtilis* ATCC 9524 (BS), *Pythium ultimum* BCCM 16,164 (PU), and *Mucor ramannianus* NRRL 1829 (MR) using the paper filter disk technique.

The purified D_1_ compound showed only antibacterial activity whereas the purified D_2_ compound showed both the ability to solubilize mineral phosphate of TCP and RP (Figure 2) as well as to inhibit the growth of all tested bacterial and fungal strains (Table 1).

**Figure 3:**
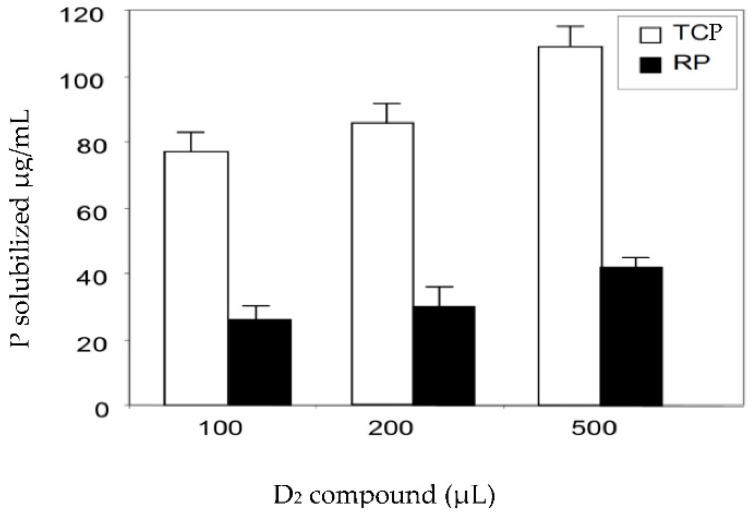
Phosphate released from TCP (4 mg/mL, white histograms) and RP (1 mg/mL, black histograms) incubated for five days at 30 °C on a rotary shaker (180 rpm) in the presence of 100, 200, and 500 µL of purified compound D_2_ or an equivalent volume of sterile bi-distilled water (negative control). The amount of soluble phosphate released from RP and TCP of the negative control was subtracted from that present in the assays containing the purified compound D_2_. The final values are arithmetic means of three independent assays and error bars represent standard deviations of the mean values of the triplicates.

Larger volumes of the pure active D_2_ fraction led to a higher release of solubilized phosphate from TCP and RP. For instance, 100 µL and 500 µL of the D_2_ fraction resulted in concentrations of solubilized phosphate of 28.6 µg/mL and 45.2 µg/mL from RP and 78.6 µg/mL and 110.7 µg/mL from TCP, respectively (Figure 3).

### 2.4. Structural Determination of the Bio-Active Compound of Fraction D_2_

After purification, the structure of the active D_2_ compound (30 mg) was elucidated by different spectroscopic methods. The HR-ESIMS and ^13^C NMR spectra revealed that the molecular formula of the D_2_ compound was C_21_H_12_O_9_N_3_Fe. The ESIMS spectrum obtained in the full-can mass range (*m*/*z*) 100–2000 and in the negative mode showed two main peaks at *m*/*z* 506 (Calcd, 506.1875) and at *m*/*z* 1035, corresponding to (M–H)^−^ and (2M + Na–2H)^−^, respectively. Table 2 summarizes the assignments of protons and carbons of the NMR spectrum of the D_2_ compound.

**Figure 4:**
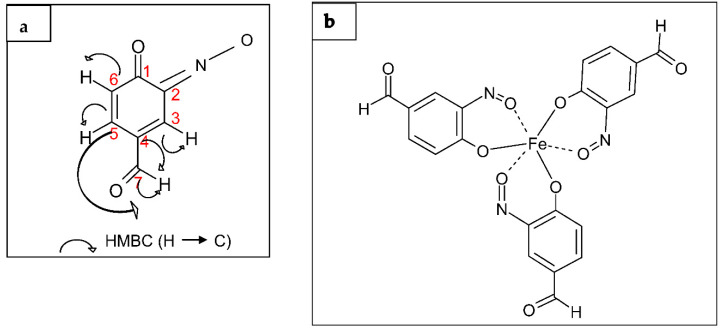
Structure and proposed mode of action of the viridomycin-like D_2_ compound (**a**) Monomer of the viridomycin-like D_2_ compound and HMBC correlation. (**b**) Proposed mode of action of the compound as an iron chelator.

Analysis of ^13^C NMR spectra and ^1^H NMR spectra revealed the presence of a ketone group and an aldehyde group and three ‘potential’ aromatic protons on the same moiety. According to its atomic composition, to the NMR data, and to the green color of its chromophore moiety, we identified this compound as a 3-imino -4-oxo- cyclohexa-1,5-diene-carbaldehydo residue. This compound is likely to constitute a monomer of the D_2_ compound (Figure 4a).

The δ values and coupling patterns of four protons on the cyclohexane ring of the chromophore (Table 2) and the long-range couplings between the aldehyde proton and the ring carbons indicated that an aldehyde group was present at C-7 of the monomer (Figure 4a). The monomer should also have a ketone group at C-1 and an oxim group (-C=N-O) at C-2 to be consistent with the molecular formula of D_2_ (Figure 4a).

The result obtained with mass spectrometry and various NMR measurements (COSY, HSQC, HMBC, NOESY, DOSY) revealed that the structure of the D_2_ compound was identified as Tris (3-imino -4- oxo- cyclohexa-1,5-diene-carbaldehydo) iron (III) as shown in Figure 4b. This compound belongs to the viridomycin family and is similar to viridomycins A, E, and F.

The greenish color of this compound suggested that it may contain iron. The presence of iron in this viridomycin-like molecule was thus tested and confirmed by the atomic absorption spectrophotometer method. The iron concentration was 0.26 mg of Fe^3+^/mg of purified viridomycin, corresponding to approximately 2 moles of iron per mole of viridomycin.

## 3. Discussion

In this issue we demonstrated that a *Streptomyces griseus*-related strain that possesses multiple plant growth-promoting activities [10,11,12] produces a molecule of the viridomycin family [17] able to solubilize RP [11,13]. This family of molecules produced by some *Actinobacteria* was discovered in 1964 under the form of greenish pigments [18] constituted by a mixture of several molecules showing subtle structural differences and thus named viridomycins (A, B, C, D, E, and F) [19,20,21]. These molecules showed weak antibiotic activity that might be due to their ability to chelate iron, a metallic ion indispensable for microbial growth. Their production was shown to be triggered under the condition of phosphate limitation [18‒21] but not under the condition of iron limitation [22]. This suggested that the first function of these molecules was to scavenge phosphate under the condition of phosphate limitation. However, our report is the first one to clearly demonstrate the ability of a molecule belonging to the viridomycin family to solubilize insoluble mineral phosphates. The capture by molecules of the viridomycin family of the positively charged counter ions of the P component of RP and TCP (including calcium, iron, etc…), is thought to destabilize the structure of RP or TCP, allowing the liberation and solubilization of the negatively charged phosphates.

Some other microorganisms, besides *Actinobacteria* [23,24,25], including *Aspergillus niger* [26], *Enterobacter* sp., *Erwinia* sp. [27], and *Penicillium rugulosum* [28] were also reported to solubilize insoluble RP by excretion of chelating substances able to form stable complexes with phosphorus counter-ions such as Al, Fe, and Ca. These results were further supported by Narsian and Patel [29], who tested the effects of known chelators including EDTA, DTPA, NTA, aluminon, and oxine on RP solubilization. However the structure of these natural chelating molecules has never been characterized [30,31]. Furthermore, these metal chelating molecules/siderophores may act as antibiotics since they chelate metals, such as iron, that are indispensable for microbial growth [32,33].

We previously demonstrated that the introduction in greenhouse soil of the *Streptomyces griseus*-like strain producing metabolites of the viridomycin family had a positive effect on plant growth and fitness and was able to limit the detrimental effects of phytopathogen fungi [12,13]. Considering its interesting properties, the spreading of this strain or of strains with similar interesting properties in greenhouse and/or open air agricultural soils could contribute to the development of more sustainable agriculture since the molecules produced by these bacteria are indeed able to limit the growth of various plant pathogens as well as to make available for plant nutrition the phosphate present in poorly soluble ground rock phosphate used as a cheap amendment in traditional agriculture.

## 4. Materials and Methods

### 4.1. Strain and Culture Conditions

Spores of the *Streptomyces griseus*-related strain isolated from a Moroccan phosphate mine [10,11,12,13] were obtained from cultures grown on solid synthetic minimal medium [10] and stored in 20% sterile glycerol at −20 °C. These spores were used to inoculate (at 10^6^ spores/mL) a 3 L culture in a liquid synthetic minimal medium (glucose 1%, NaNO_3_ 0.2%, MgSO_4_·7 H_2_O 0.05%, KCl 0.05%, FeSO4·7 H_2_O 0.001%, pH 7.2) supplemented with 0.1% of washed RP as a sole phosphate source. Cultures were grown for five days at 28 °C under constant agitation on a rotary shaker at 180 rpm. The rock phosphate used in this study is a calcium hydroxyapatite originating from the Khouribga phosphate mine (Morocco) constituted by O, 56.53%; Ca, 16.35%; P, 9.37%; F, 2.42%; Al, 2.03%; Mg, 1.94%; Na, 1.81%; S, 0.77%; Fe, 0.60%, and Sn, 0.12% [10].

### 4.2. Production and Extraction of Active Compounds

The culture mentioned above was centrifuged at 10,000× *g* for 10 min. The collected supernatant was filtered through a 0.45 µm-pore-size filter (Supor-450; Pall Corporation, Port Washington, NY, USA) to remove cell debris. The filtrate was extracted with isoamyl alcohol (3-methyl butan-1-ol, Sigma, St. Quentin Fallavier Cedex, France), 100: 25 (*v*/*v*) acidified to pH 2 with HCl 6 N. The greenish organic phase was collected and concentrated to dryness (1.65 g) under vacuum on a Rotavapor (Laborata 4000, Heidolph) at 40 °C and then dissolved in 2 mL of MeOH to obtain the crude extract called E (275 mg/mL).

### 4.3. Detection and Purification of Active Fractions with Antimicrobial Activities

In order to assess the presence of different bio-active metabolites in the crude extract by bio autography [34], volumes of 40 μL and 60 μL of the extract were spotted onto 20 × 20-cm silica gel plates (Merck Art. 5735, Kiessel gel 60F254) in order to assess its inhibitory activity against the Gram positive bacterium *Bacillus subtilis* ATCC 9524 and the fungus *Pythium ultimum* BCCM 16164, respectively. The TLC plates were developed using a mixture of ethyl acetate–methanol, 100: 15 (*v*/*v*) and were air-dried overnight at 37 °C to remove the solvents. After migration, the separated compounds were visualized at 254 nm under UV light (absorbance) and at 365 nm (fluorescence). The TLC plates were then placed in a plastic bioassay dish (23 × 23 × 2.2 cm^3^, Fisher Scientific Labosi) and overlaid with 150 mL of nutrient agar or PDA media (containing 7 g/L of agar), inoculated with *B. subtilis* (10^6^ cfu/mL) or *P. ultimum* (100 cfu/mL), respectively. Once the agar solidified, the plate was incubated at 30 °C. Four spots with bio-activity were detected by bio autography on the TLC plates. The four fractions designated as A, B, C, and D showed Rf values of 0.9, 0.45, 0.37, and 0.3, respectively. After 24 h of incubation for *P. ultimum* and 48 h for *B. subtilis*, clear areas corresponding to zones of growth inhibition of the microorganisms were detected, indicating the location of antibiotic compounds on the TLC plates. A and D spots showed both antibacterial and antifungal activities against the tested micro-organisms, while B and C showed only antibacterial activity.

Subsequently, the crude extract (E, 275 mg/mL) was deposited on preparative silica gel plates in order to collect the A and D fractions in sufficient quantity. We obtained 115 mg/mL of fraction A (41.8% of total E) and 98.5 mg/mL of fraction D (35.8% of total E).

#### 4.3.1. Antimicrobial Bioassay against Bacteria and Fungi

The active fractions were collected and their antimicrobial activities were tested against two bacterial (*Bacillus subtilis* ATCC 9524 and *Micrococcus luteus* ATCC 381) and fungal (*Pythium ultimum* BCCM 16,164 and *Mucor ramannianus* NRRL 1829) species using the paper filter disk technique: To do so, 20 µL of the active purified fractions were deposited on sterile cellulose disks (5 mm diameter, Pasteur Institute) and allowed to dry overnight at 37 °C to evaporate the methanol solvent. Subsequently they were placed aseptically on the surface of plates of nutrient agar (Difco Maryland, USA) or of Sabouraud agar medium (Biorad, Schiltigheim, France) inoculated with bacteria at 10^6^ cfu/mL and fungi at 100 cfu/mL, respectively. Sizes of the inhibition zones were determined after 24 h of incubation for *Bacillus subtilis* ATCC 9524 and *Micrococcus luteus* ATCC 381 and 48 h for *Pythium ultimum* BCCM 16,164 and *Mucor ramannianus* NRRL 1829. Controls consisted of sterile cellulose discs. Three replicates were performed for each fraction for each microorganism.

#### 4.3.2. Determination of Phosphate-Solubilizing Abilities

In order to check the phosphate-solubilizing abilities of the active antimicrobial fractions, various volumes of the selected fractions or of sterile distilled water (negative control) were added to 1 mL of sterile bi-distilled water containing 1 g/L of RP or 4 g/L of TCP (Sigma Aldrich, St. Quentin Fallavier Cedex, France). The mixtures were incubated at 30 °C for five days on a rotary shaker (180 rpm). The values obtained with the negative controls were subtracted from the values obtained with the fractions. The amount of soluble phosphate released from RP and TCP was estimated by the Olsen and Sommers method [35]. The final values are the arithmetic mean of three independent assays (Figure 3). The only fraction with both antibiotic and phosphate-solubilizing activities was fraction D that was selected for further investigations.

### 4.4. Purification and Structure Elucidation of the Active Compound

The active fraction D was purified by HPLC (Waters: controller 600, pump 600, dual *λ* absorption detector 2487, Linear Recorder); column C18 (250 × 7.8 mm^2^ UP ODS); mobile phase: linear gradient of methanol–H_2_O from 0 to 100% for 52 min; flow rate: 1 mL/min, *λ* detection at 220 nm and at 420 nm. The column temperature was 30 °C. The injection volume was 200 µL. Under these conditions, the retention time was recorded at 17.6 min. This fraction was shown to contain two main peaks, D_1_ and D_2_, but only D_2_ showed both phosphate-solubilizing and anti-microbial activities.

After purification of the active compound present in D_2_ by HPLC, the latter was subjected to spectroscopic studies. ^1^H NMR spectroscopy: Varian Unity 500 (500 MHz), Bruker AMX 500 (500 MHz), Varian Inova 500 (500.33 MHz). ^13^C NMR spectroscopy: Varian Unity 500 (125.8 MHz), Varian Inova 500 (125.8 MHz). Chemical shifts were measured relative to tetramethylsilane as an internal standard. The homonuclear and heteronuclear 1D and 2D NMR spectra were recorded on a Varian Inova 500 instrument. Mass spectrometry was performed with an LCC ion-trap mass spectrometer (INSERM, Purpan, Toulouse). Samples were analyzed by electrospray ionization in both negative and positive ion mode, and the full-scan mass range (*m*/*z*) was 100–2000.

### 4.5. Determination of the Iron Content of the Active Compound

The presence of iron in the purified D_2_ compound was verified as follows. Laboratory glassware needed was kept overnight in a 5% nitric acid solution before use and then rinsed with triple-distilled water and dried in an oven. Subsequently, 3 mg of the purified product was dissolved in 5 mL triple-distilled water acidified with 5% of nitric acid and heated for 30 min at 60 °C. The iron concentration was determined as described in Rauret et al. [36] and measured at 248.3 nm using a Unicam SP 969 atomic absorption spectrophotometer. A standard curve was established with a solution of Fe^3+^ (FeCl_3_, Sigma) in the 0.06 to 5 mg/L range.

## Figures and Tables

**Table 1:** Antimicrobial activity of the D_2_ compound.

	Antimicrobial Activity ^a^ against			
	Gram Positive Bacteria		Fungi	
	*Bacillus subtilis* ATCC 9524	*Micrococcus luteus* ATCC 381	*Pythium ultimum* BCCM 16164	*Mucor ramannianus* NRRL 1829
Diameter of the inhibition zone (mm)	29 ± 1.2	45 ± 1.4	27 ± 1.1	38 ± 1.0

^a^: Activity assessed by the agar diffusion method (filter disc i.d. 6 mm, c = 20 µg/mL, 20 µL/disc).

**Table 2:** NMR Spectroscopic Data (500 MHz, CD_3_CN) of the D_2_ Compound.

Position	δ_C_, Type	δ_H_ (J in Hz)	HMBC ^a^
1	182.0		
2	159.3		
3	115.7	7.56 s	Observed
4	123.9		Observed
5	134.5	7.96 d (9)	Observed
6	122.5	7.16 d (9)	
7	190.5	9.78 s	Observed

Coupling constants in Hertz are given in parentheses. ^a^: HMBC = heteronuclear multiple bond correlation.

## Data Availability

The data supporting the findings of this study are available from the corresponding author.
